# Supplementary material for: Site-specific phosphorylation and caspase cleavage of GFAP are new markers of Alexander disease severity
Source: eLife. 2019 Nov 4;8:e47789. doi: 10.7554/eLife.47789 (PMC6927689; doi:10.7554/eLife.47789)
Supplement: Supplementary file 1. [file elife-47789-supp1.docx]

**Supplementary File 1 |** Donor information for AxD post-mortem human brain specimens.

| **ID number** | **age of death (years)** | **age of death (days)** | **sex** | **GFAP Mutation** | **PMI (hours)** | Race | Cause of Death | Other references |
| --- | --- | --- | --- | --- | --- | --- | --- | --- |
| 1482 | 0 | 244 | Female | D395Y | 2 | Caucasian | Complication of disorder |  |
| 1070 | 0 | 347 | Male | R239H | 4 | Caucasian | Complication of disorder | Ann Neurol, 2005, patient # 23 |
| 885 | 0 | 192 | Female | E373K | 18 | Caucasian | Complication of disorder | Ann Neurol, 2005, patient # 37 |
| 5488 | 1 | 0 | Female | R239H | 7 | Caucasian | Complication of disorder |  |
| 1161 | 2 | 175 | Male | R239C | 4 | Caucasian | Complication of disorder |  |
| 2768 | 2 | NA | Female | N77S | 17 | Caucasian | Complication of disorder |  |
| 338 | 6 | 87 | Male | R239C | 12 | Caucasian | Complication of disorder |  |
| 613 | 13 | 364 | Male | R79C | 7 | Caucasian | Complication of disorder | Nature Genetics, 2001, patient # 1 |
| 5377 | 27 | 139 | Female | K63E | 22 | Caucasian | Complication of disorder |  |
| 5517 | 28 | 245 | Female | R79C | 18 | Caucasian | Complication of disorder |  |
| M3596 | 33 | 273 | Female | E210K | 20 | Caucasian | Complication of disorder | Ann Neurol, 2005, patient # 13 |
| 5109 | 42 | 217 | Female | D417A | 4 | Caucasian | Complication of disorder |  |
| 4858 | 50 | 139 | Female | S247P | 17 | Caucasian | Complication of disorder |  |
