# Supplementary material for: Site-specific phosphorylation and caspase cleavage of GFAP are new markers of Alexander disease severity
Source: eLife. 2019 Nov 4;8:e47789. doi: 10.7554/eLife.47789 (PMC6927689; doi:10.7554/eLife.47789)
Supplement: Supplementary file 2. [file elife-47789-supp2.docx]

**Supplementary File 2.** Donor information for control (non-AxD) post-mortem human brain specimens.

| **ID number** | **age of death (years)** | **age of death (days)** | **sex** | **GFAP Mutation** | **PMI (hours)** | Race | Cause of Death |
| --- | --- | --- | --- | --- | --- | --- | --- |
| 1547* | 1 | 259 | Male | N/A – Control | 10 | African American | Asthma |
| 5941 | 2 | 0 | Male | N/A – Control | 9 | Hispanic | Drowning |
| 103 | 2 | 75 | Female | N/A – Control | 11 | African American | Meningitis |
| 1791* | 2 | 286 | Female | N/A – Control | 12 | African American | Drowning |
| 4670 | 4 | 237 | Male | N/A – Control | 17 | Caucasian | Commotio Cordis |
| 4898 | 7 | 272 | Male | N/A – Control | 12 | Caucasian | Accident, Drowning |
| 1706 | 8 | 214 | Female | N/A – Control | 20 | African American | Rejection of Cardiac Allograft Transplantation |
| 1670 | 13 | 99 | Male | N/A – Control | 5 | Caucasian | Asphyxia By Hanging |
| 1711* | 27 | 340 | Female | N/A – Control | 4 | Caucasian | Car accident, head and neck injuries |
| 1011 | 29 | 305 | Male | N/A – Control | 4 | African American | Head injuries |
| 632 | 34 | 71 | Male | N/A – Control | 6 | Caucasian | Accident, Multiple Injuries |
| 4640 | 47 | 124 | Female | N/A – Control | 5 | Caucasian | Pneumonia |
| 4915 | 49 | 160 | Male | N/A – Control | 5 | Caucasian | ASCVD |

*denotes samples used in mass spec analysis
