# Supplementary material for: Site-specific phosphorylation and caspase cleavage of GFAP are new markers of Alexander disease severity
Source: eLife. 2019 Nov 4;8:e47789. doi: 10.7554/eLife.47789 (PMC6927689; doi:10.7554/eLife.47789)
Supplement: Supplementary file 3. [file elife-47789-supp3.docx]

**Supplementary File 3 |** Summary from off-target sequencing from CRISPR/Cas9 editing.

| **Gene name** | **Gene ID** | **AxD patient iPSCs** | **MDCL14**  **(CRISPR control)**  **iPSCs** | **MDCL11**  **(isogenic control) iPSCs** |
| --- | --- | --- | --- | --- |
| MRNIP/SQSTM1 | 51149/8878 | 1bp insertion of G in 3’UTR (chr5. 179840448) | 1bp insertion of G in 3’UTR (chr5. 179840448) | 1bp insertion of G in 3’UTR (chr5. 179840448) |
| COMT | 1312 | Silent mutation CAC 🡪CAU (chr22.19962712) | Silent mutation CAC 🡪CAU (chr22.19962712) | Silent mutation CAC 🡪CAU (chr22.19962712) |
| GNB1L/TBX1 | 54584/6899 | No mutations | No mutations | No mutations |
| INPP4B | 8821 | No mutations | No mutations | No mutations |
| POLD1 | 5424 | No mutations | No mutations | No mutations |
| RABEP2 | 79874 | Mutation outside of exon A 🡪G (chr16.28906323) | Mutation outside of exon A 🡪G (chr16.28906323) | Mutation outside of exon A 🡪G (chr16.28906323) |
| SGSM3 | 27352 | No mutations | No mutations | No mutations |
| WNT5B | 81029 | Mutation outside of exon A🡪C (chr12.1639668) | Mutation outside of exon A🡪C (chr12.1639668) | Mutation outside of exon A🡪C (chr12.1639668) |
| DMPK | 1760 | No mutations | No mutations | No mutations |
