# Supplementary material for: Site-specific phosphorylation and caspase cleavage of GFAP are new markers of Alexander disease severity
Source: eLife. 2019 Nov 4;8:e47789. doi: 10.7554/eLife.47789 (PMC6927689; doi:10.7554/eLife.47789)
Supplement: Supplementary file 4. [file elife-47789-supp4.docx]

**Supplementary File 4** | GFAP phosphorylation motifs and candidate kinases.

| **Position** | **Sequence** | **Motif (red=phospho site)** | **Features of motif described in the literature** |
| --- | --- | --- | --- |
| 11-13 | RRS | RXpS | PKA kinase substrate motif |
| 11-13 | RRS | [R/K]X[pS/pT] | PKA kinase substrate motif |
| 11-13 | RRS | [R/K]X[pS/pT] | PKC kinase substrate motif |
| 12-17 | RSYVSS | X[pS/pT]XXX[A/P/S/T] | G protein-coupled receptor kinase 1 substrate motif |
| 13-16 | SYVS | [pS/pT]XX[S/T] | Casein Kinase I substrate motif |
| 13-16 | SYVS | pSXX[E/**pS*/pT***] | Casein Kinase II substrate motif |
| 13-16 | SYVS | [pS/pT]XX[E/D/**pS*/pY***] | Casein Kinase II substrate motif |
| 13 - 17 | SYVSS | pSXXX[pS/pT] | MAPKAPK2 kinase substrate motif |
| 13 - 17 | SYVSS | pSXXX**pS*** | GSK3 kinase substrate motif |
| 16 - 19 | SSGE | pSXX[E/D] | Casein kinase II substrate motif |
| 16 - 19 | SSGE | [pS/pT]XX[E/D] | Casein Kinase II substrate motif |
| 16 - 19 | SSGE | [pS/pT]XX[E/D] | Casein Kinase II substrate motif |
| 17 - 19 | SGE | pSX[E/**pS*/pT***] | Casein Kinase II substrate motif |
| *****indicates the residue that has to be phosphorylated already for the enzyme to recognize the motif  PhosphoMotif Finder was used to generate the motif predictions (Amanchy et al. *Nat Biotechnology* 2007) | | | |
